# Importance of Ophthalmic Nursing in Primary Healthcare Systems

**Published:** 2016

**Authors:** Mohammad MORADI

**Affiliations:** Medical School, Shahed University, Tehran, Iran

**Keywords:** Ophthalmic Nursing, Primary Healthcare, Specialized Nursing-care, Cost-Effective

## Specialized nursing care

We have arrived at a new era in nursing care. Nurses are now active in both hospital-based healthcare and community health initiatives (1). Community and healthcare provider expectations have increased over the past 20 years largely because of great advances in technology, new healthcare delivery systems, and changing priorities. Advanced practice educational programs include training and developing nurse practitioners to be competent and independent in running their own office, which includes starting their practice and providing care to their own patients. Therefore, specialized nurses need to adjust, expand, and integrate their practical skills to respond to the growing demands and expectations of patients, individuals, and decision makers (2). An ideal specialty nurse should be able to make clinical decisions based on modern medicine and technology considering patient welfare. Decisions should also be consistent with the nature of patients being cared. Trainees in specialized nursing programs are generally registered nurses who wish to increase their level of knowledge, expertise, and experience to provide appropriate services and to meet the changing needs of healthcare consumers (3).

## Role of ophthalmic nurses in the advancement of visual care

Ophthalmic nurses can assess a patient’s visual potential and his or her capability to function independently. Ophthalmic nurses also help patients overcome psychological obstacles and provide necessary assistance to increase patient independence. Ophthalmic nurses can educate patients on and increase awareness of public resources and available services for the visually impaired (2-3).

The future of ophthalmic nursing depends on the cultural, social, and financial background of each community. The most prominent feature unique to ophthalmic nurses is their ability to apply medical and nursing interventions to fulfill visual care requirements. While physicians diagnose diseases, prescribe medication, and perform surgeries, nurses help patients adapt to new and persistent lifestyle-altering conditions (4). Therefore, ophthalmic nurses should commit to dedicating themselves to continued learning, which is necessary to develop and maintain the highest standard of care. This should be facilitated by nurses themselves and overseen by program developers and others involved in multidisciplinary group care.

The main objective of specialized nursing educational processes is to train nurses to be quick, logical thinkers, even under pressure and during emergency situations. Their decisions should be based on the compilation, assessment, and utilization of known information to accurately evaluate a situation and determine the best course of action (5). For example, ophthalmic nurses should collaborate with professional organizations to gain better access to and awareness of resources for patients. It is obvious that ophthalmic nurses play an important role as teachers and advisors to patients and healthcare providers through development, learning, and communication of multidisciplinary team impressions and ophthalmic knowledge. Ophthalmic nurses are ideal for this because of the intimate and close relationships they develop with patients, which allows nurses to better understand patient needs and demands. This helps patients gain independence and the ability to care for themselves.

International organizations have developed a competency framework based on knowledge, ability, and interventions that significantly improve the likelihood of an ophthalmic nurse having a successful career. This framework defines the following three levels of clinical nurses: a competent nurse (Level 1), an experienced/adept nurse (Level 2), and a senior practitioner/expert nurse (Level 3). This level structure guides ophthalmic nurses in the progression of their careers in the clinical setting, serves as an example for nurses delivering ophthalmic care in other settings, and provides guidelines for healthcare providers to know the level of competency of each ophthalmic nurse. It can also be used as a framework for educational programs, bridge the gap between classroom education and clinical practice, and make nursing care in the ophthalmic sector more consistent. Moreover, providing a model for navigating the continually evolving and extending field of ophthalmic nursing is helpful for healthcare professionals in primary healthcare and visual rehabilitation because it establishes standards for professional progression. We hope that this level framework will be widely adopted by nurses in ophthalmic healthcare systems.

## Responsibilities

Responsibilities of ophthalmic nurses include evaluating, diagnosing, treating, and discharging patients with ocular conditions and diseases/disorders. They also manage patients referred from general practitioners and primary healthcare units, perform initial screening, monitor disease progression, and assist with early treatment of chronic ocular conditions (e.g., glaucoma, diabetic retinopathy, and dry eye). Regarding surgical care, ophthalmic nurses perform primary, follow-up, and discharge assessments of patients who have undergone surgery for cataract and other ocular diseases/disorders. Ophthalmic nurses also assist in managing children and adults with ocular conditions, functioning as both an educator and a practitioner. These responsibilities allow them to update their practical experience, while educating others involved in ophthalmic nursing and ophthalmic healthcare duties. These duties include performing minor interventions without supervision (e.g., minor procedures and adnexal surgery) and assisting in ophthalmic surgeries, including YAG laser capsulotomy (2-5). 

## Future of ophthalmic nursing

Ophthalmic nurses play a key role throughout the entire domain of ophthalmic health. Many studies have confirmed a positive correlation between nurse competency and patient care quality (6). Ophthalmic nurses contribute to cost-effective health care, which is of high priority to both government-funded and privately funded institutions. If the visibility of this career path is increased, the field would attract more candidates and result in a higher level of patient trust, both of which would pave the way for better healthcare systems. It has been suggested that training courses dedicated to developing competent ophthalmic nurses and public seminars introducing this important healthcare field would be beneficial to the healthcare systems.

## DISCLOSURE

### Conflicts of Interest:

None declared.

No funding or sponsorship was received for this study. All the aforementioned authors met the International Committee of Medical Journal Editors (ICMJE) criteria for authorship for this manuscript, take responsibility for the integrity of the work as a whole, and have provided approval for the revised manuscript to be published.
